# Discrepancies Between Pathological Distinction and DNA Genotyping in the Diagnosis of Hydatidiform Moles

**DOI:** 10.7759/cureus.85953

**Published:** 2025-06-13

**Authors:** Yuri Hasegawa, Koh Nagata, Shoko Miura, Ai Nagata, Hiroyuki Mishima, Akira Kinoshita, Koh-Ichiro Yoshiura, Kiyonori Miura

**Affiliations:** 1 Obstetrics and Gynecology, Nagasaki University Graduate School of Biomedical Sciences, Nagasaki, JPN; 2 Human Genetics, Nagasaki University Atomic Bomb Disease Institute, Nagasaki, JPN; 3 Human Genetics, Leading Medical Research Core Unit, Nagasaki University Graduate School of Biomedical Sciences, Nagasaki, JPN

**Keywords:** complete hydatidiform mole, distinction, dna genotyping analysis, p57kip2 immunohistochemistry, partial hydatidiform mole

## Abstract

Introduction

The incidence of invasive moles and choriocarcinoma after uterine evacuation differs between a complete hydatidiform mole (CHM) and a partial hydatidiform mole (PHM). Accurately distinguishing between these two types is important. This study aimed to investigate diagnostic differences in hydatidiform moles using p57kip2 immunohistochemistry and DNA genotyping analysis in trophoblastic tissue.

Materials and methods

Twenty-four patients who underwent uterine evacuation or total hysterectomy for suspected hydatidiform moles between 2013 and 2022 were included in this study. Pathologists performed p57kip2 immunohistochemistry in addition to hematoxylin and eosin staining. A DNA genotyping analysis was performed on hydatidiform villus tissue and blood samples from patients and their partners (if possible) to differentiate between CHM and PHM.

Results

Of the 16 CHM cases diagnosed histologically, one was diagnosed as decidua by DNA genotyping. Of the eight PHM cases diagnosed histologically because of positive p57kip2 immunohistochemistry, four were diagnosed as CHM by DNA genotyping, and two were diagnosed as decidua. The sensitivity, specificity, positive predictive value, and negative predictive value of the pathological diagnosis of the 21 cases, excluding the three cases diagnosed as decidua by DNA testing, were 100%, 33%, 71.4%, and 28.6%, respectively (P = 0.0183, Pearson’s chi-square test). Of the 24 cases, blood samples were obtained from the partners in 15 cases. Of the 15 cases, 12 were homodisomy and three were heterodisomy.

Conclusions

Hematoxylin and eosin staining and p57kip2 immunohistochemistry are useful tools for differentiating CHM from PHM, but combining them with DNA genotyping analysis leads to a more accurate and reliable diagnosis.

## Introduction

An accurate diagnosis of a complete hydatidiform mole (CHM), a partial hydatidiform mole (PHM), and early miscarriage that is not a hydatidiform mole is important because the incidence of invasive moles and choriocarcinomas developing after evacuation is different. The incidence of secondary invasive moles after evacuation was reported to be 10-20% for CHM and 2-4% for PHM [1,2], while the incidence of choriocarcinoma was 1-2% for CHM and even lower for PHM [1,2].

Histologically, typical CHMs are characterized by enlarged, edematous villi with notable circumferential trophoblastic hyperplasia, central cistern formation, cytological atypia, and trophoblastic inclusion. In contrast, morphological features of PHM show two populations of villi (large, irregular, hydropic villi and small fibrotic villi) with irregular scalloping, trophoblastic inclusions, and mild trophoblastic hyperplasia [3-5]. Despite these morphological differences, CHM and PHM share many morphological characteristics, which causes difficulty in distinguishing between these two conditions pathologically.

Genetic classification is useful when the histological distinction between CHM and PHM is difficult. CHM chromosomes are defined as having two sets of chromosomes that are derived paternally (i.e., androgenic origin) [6-8]. However, PHM is often a triploid of a paternal haploid and maternal diploid, and this characteristic can be used to distinguish CHM from PHM using DNA genotyping. Tumor-suppressing subtransferable candidate 3 (TSSC3) and p57kip2 are expressed exclusively from the maternal allele on the imprinted gene cluster of chromosome 11, 11p15.55. Therefore, immunostaining positive for p57kip2 or TSSC3 indicates the existence of the maternal allele [9,10]. In villous tissue, the paternally derived alleles of these genes are transcriptionally suppressed by epigenetic modifications including methylation. Additionally, there is no protein expression of p57kip2 [9] and TSSC3 [10] in cytotrophoblastic and chorionic stromal cells of CHM, only with paternal alleles. Although immunohistochemical staining is useful in differentiating between CHM and PHM [4,11,12], 20-30% of cases are misclassified, even after morphological evaluation by well-trained gynecological pathologists and even with p57 immunohistochemical examination.

CHM can develop through uniparental paternal homo- or heterodisomy. Some reports have shown that the risk of developing gestational trophoblastic disease (GTD) after CHM treatment is higher in heterodisomy than in homodisomy [13-15]. However, other reports have shown that the risk of developing GTD is the same in both of these groups [16].

This study aimed to clarify the extent to which the diagnosis of CHM and PHM differs between these two types by performing immunohistochemical tests and by DNA genotyping, and to investigate the onset rate of GTD after treatment of homo- or heterodisomic CHM.

## Materials and methods

Sample collection

All patients who participated in this study attended Nagasaki University Hospital between January 2013 and December 2022. They were diagnosed with suspected trophoblastic disease using ultrasonography and underwent uterine curettage or total hysterectomy at Nagasaki University Hospital. Samples were taken from the cystic chorionic tissue of the uterine contents, and a pathological examination and DNA polymorphism analysis were performed. The uterine contents, presumed to be chorionic villi, were selected macroscopically (although non-villous tissue was also included). The collected tissue was divided for pathological examination and DNA analysis. The samples for DNA analysis were temporarily preserved in saline, quickly homogenized, and DNA was extracted (the DNA extraction method is described below). A blood sample for serum beta-human chorionic gonadotropin concentration measurement was obtained from the patient immediately prior to the uterine evacuation procedure. Additionally, blood samples (7 mL) from the patients were collected in tubes with ethylenediaminetetraacetic acid before the evacuation. In this study, to prevent contamination of the chorionic tissue and decidua, chorionic tissue was collected during surgery, and peripheral blood was used to represent maternal elements. In addition, if possible, we also analyzed the blood of the patient’s partner to determine whether the fertilization was by one sperm (resulting in homodisomy in CHM) or two sperm (which could result in heterodisomy in CHM) using DNA polymorphism analysis.

Pathological examination

The collected chorionic tissue was fixed in formalin, embedded in paraffin, and sections were cut and then stained with hematoxylin and eosin. In addition, immunohistological staining using p57kip2 was performed following a standard procedure. Immunohistochemical staining for p57kip2 was performed using a mouse monoclonal antibody (clone 57P06, MS-1062-R7, Thermo Fisher Scientific, Waltham, MA) on formalin-fixed, paraffin-embedded tissue sections. Staining was carried out on the fully automated Leica BOND-III system (Leica Biosystems, Nussloch, Germany) using the BOND Polymer Refine Detection Kit. Tissue sections were deparaffinized and subjected to antigen retrieval with BOND Epitope Retrieval Solution 2 (ER2; ethylenediaminetetraacetic acid (EDTA)-based buffer, pH 9.0) at 100°C for 20 minutes. Following antigen retrieval, endogenous peroxidase activity was blocked using peroxide block for five minutes. The primary antibody was applied at a 1:2 dilution and incubated at room temperature for 15 minutes. This was followed by an eight-minute incubation with post-primary reagent and an additional eight-minute incubation with polymer-based secondary antibody. Immunoreactivity was visualized using 3,3'-diaminobenzidine (DAB) as the chromogen for 10 minutes, and sections were counterstained with hematoxylin for five minutes.

A pathological diagnosis was made by a well-trained pathologist certified by the Japanese Society of Pathology who did not know the results of the DNA genotyping analysis.

DNA genotyping analysis

DNA was extracted from trophoblastic tissue and peripheral blood samples of the patient and their partner using a QIAamp DNA Blood Mini Kit (Qiagen, Inc., Valencia, CA). The extracted DNA was amplified with fluorescent-labeled primers for a panel of 15 short tandem repeat markers (AmpFLSTR Identifiler PCR Amplification Kit, Applied Biosystems, Foster City, CA). The loci markers were D8S1179, D21S11, D7S820, CSF1PO, D3S1358, TH01, D13S317, D16S539, D2S1338, D19S433, vWS, TPOX, D18S51, D5S818, and FGA amelogenin sex markers. Polymerase chain reaction amplification and capillary electrophoresis were performed on an ABI 3100 Genetic Analyzer (Applied Biosystems) by following the manufacturer’s instructions. Capillary electrophoresis data from blood and chorionic tissue samples from the patient and partner were analyzed with a fragment analyzer (GeneMapper®, Applied Biosystems) to identify alleles at each locus.

In cases where blood could be taken from the patient’s partner, CHM was diagnosed according to homodisomy or heterodisomy. In cases where the partner’s DNA was not available, hydatidiform mole was diagnosed according to whether it was CHM (i.e., without the maternal allele) or PHM (i.e., with the maternal allele).

Statistical analysis

The patients’ age and serum human chorionic gonadotropin concentrations were compared between the groups with CHM, PHM, and non-hydatidiform specimens using the Kruskal-Wallis test. The accuracy of p57Kip2 immunohistochemistry for determining hydatidiform moles was analyzed using Pearson’s chi-square test. Statistical analyses were performed using JMP Pro 16 (version 16.2.0; SAS Institute Inc., Cary, NC) with a P-value of 0.05 as the threshold for a significant difference.

## Results

Sample collection and pathological diagnosis

During the study period, 24 patients underwent evacuation or total hysterectomy for suspected hydatidiform mole. Table 1 lists the pathological diagnoses and diagnoses based on DNA genotyping for all cases. Of the 24 suspected hydatidiform moles, 16 were classified as CHM without p57kip2 expression and eight as PHM with p57kip2 expression based on the results of immunohistochemistry. Among these, cases 10 and 12 showed a re-elevation of serum human chorionic gonadotropin (hCG) levels after evacuation. Therefore, these two cases were clinically diagnosed as invasive moles and were treated with methotrexate. No secondary complications were observed in the other cases. Table 2 shows the patients’ background and pathological diagnoses. The median age of the patients was 33 years in those with CHM and 33 years in those with PHM, with no significant difference between the two groups. The median serum beta-hCG concentration before uterine evacuation was 136,358.5 mIU/mL in patients with CHM and 143,230.5 mIU/mL in those with PHM, with no significant difference between the two groups (Kruskal-Wallis test).

**Table 1:** Diagnosis based on pathological diagnosis and DNA genotyping of all cases. CHM: complete hydatidiform mole; PHM: partial hydatidiform mole; hCG: human chorionic gonadotropin.

Case	Age (years)	Serum hCG levels (mIU/mL)	p57kip2	Pathological diagnosis	DNA genotyping	Homodisomy or heterodisomy
1	27	71,608	Positive	PHM	PHM	-
2	32	190,388	Negative	CHM	CHM	Homodisomy
3	26	145,313	Positive	PHM	Decidua	-
4	29	40,084	Negative	CHM	Decidua	-
5	36	72,367	Negative	CHM	CHM	Homodisomy
6	50	146,505	Positive	PHM	CHM	Homodisomy
7	30	141,148	Positive	PHM	Decidua	-
8	34	23,072	Negative	CHM	CHM	Heterodisomy
9	44	340,688	Negative	CHM	CHM	Heterodisomy
10	40	153,906	Negative	CHM	CHM	Homodisomy
11	29	79,360	Negative	CHM	CHM	Homodisomy
12	35	451,674	Negative	CHM	CHM	-
13	18	118,811	Negative	CHM	CHM	-
14	28	180,901	Negative	CHM	CHM	Homodisomy
15	25	302,699	Positive	PHM	CHM	Homodisomy
16	36	116,319	Positive	PHM	CHM	Homodisomy
17	33	83,721	Negative	CHM	CHM	Homodisomy
18	44	54,369	Positive	PHM	PHM	-
19	25	453,838	Negative	CHM	CHM	Heterodisomy
20	29	49,357	Negative	CHM	CHM	Homodisomy
21	53	299,820	Positive	PHM	CHM	-
22	17	1,047,196	Negative	CHM	CHM	-
23	33	75,970	Negative	CHM	CHM	Homodisomy
24	50	1128602	Negative	CHM	CHM	Homodisomy

**Table 2:** Background and pathological diagnosis. CHM: complete hydatidiform mole; PHM: partial hydatidiform mole; hCG: human chorionic gonadotropin. ^a ^P < 0.05 was considered significant. The Kruskal–Wallis test was performed for the analysis.

	Pathological diagnosis (n = 25)			
	CHM (p57Kip2 immunohistochemistry: negative) (n = 16)	PHM (p57Kip2 immunohistochemistry: positive) (n = 8)	Chi-square value	P-value ^a^
Age (years old), Median (min-max)	33 (17-50)	33 (25-53)	0.377	0.54
Serum beta hCG levels (mIU/mL), Median (min-max)	136,358.56 (23,072-1,128,602)	143,230.5 (54,369-302,699)	0.094	0.76

Differences between pathological diagnosis and diagnosis using DNA genotyping

Table 3 shows the results of the pathological diagnosis by p57kip2 immunohistochemistry and DNA genotyping. Of the 16 CHM cases without p57kip2 expression, one contained a maternal component only, as shown by DNA genotyping. Of the eight PHM cases with p57kip2 expression shown by immunohistochemical staining, four were diagnosed as CHM by DNA genotyping, and two contained a maternal component only. Samples that contained a maternal component only were considered as having DNA extracted from the decidua in the evacuated mole. Although cystic villi were macroscopically sampled during chorionic villus sampling, the sample diagnosed as decidua by DNA analysis was considered to contain only maternal tissue. Therefore, they were not included in the subsequent analysis. The sensitivity, specificity, positive predictive value, and negative predictive value of the pathological diagnosis of the 21 cases, excluding the three cases with DNA extracted from the decidua, were 100%, 33.3%, 71.4%, and 28.6%, respectively (P = 0.0183, Pearson’s chi-square test). Supplementary Data shows the data for all cases.

**Table 3:** Differences between the pathological diagnosis and diagnosis by DNA genotyping analysis. Of the 16 cases diagnosed histologically as CHM, one was diagnosed as PHM by DNA genotyping. Of the eight cases diagnosed as PHM by p57kip2 immunohistochemistry, four were diagnosed as CHM by DNA genotyping. CHM: complete hydatidiform mole; PHM: partial hydatidiform mole.

		Diagnosis by DNA genotyping analysis		
		CHM	PHM	Decidua
Pathological diagnosis	CHM (p57Kip2 immunohistochemistry: negative)	16	0	1
	PHM (p57Kip2 immunohistochemistry: positive)	4	2	2

**Figure 1:**
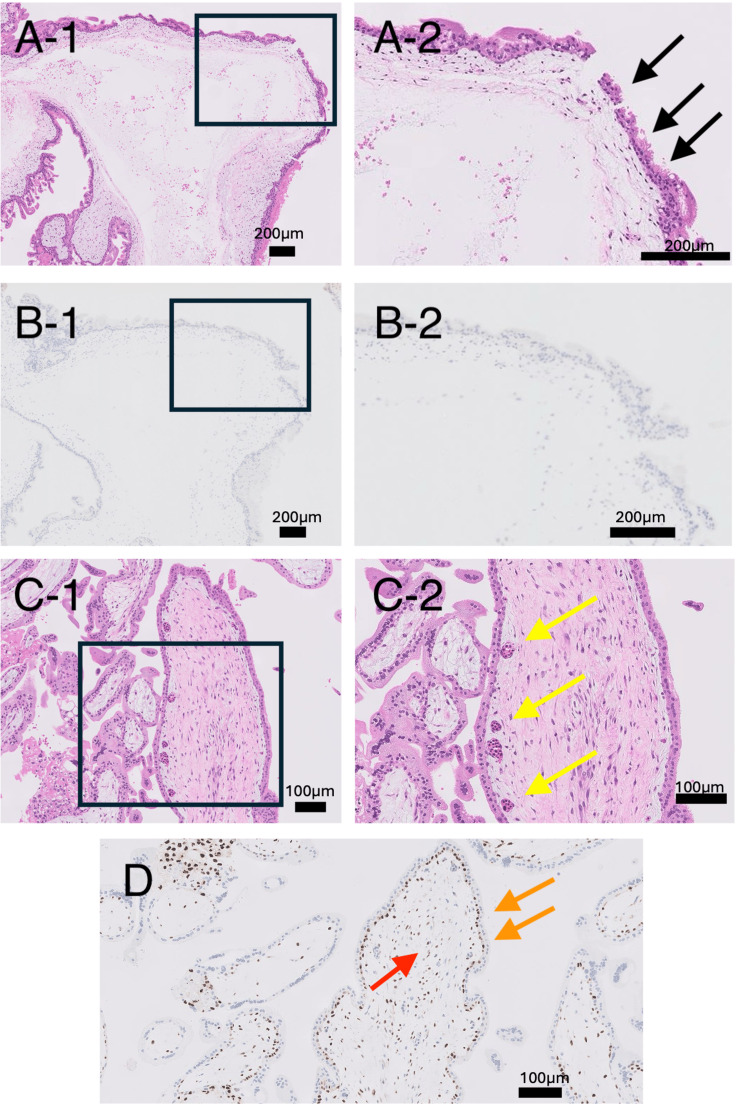
Typical pathological findings of CHM and PHM. (A) HE staining of a case of CHM (No. 23). A-1 shows a low magnification, and A-2 is a high magnification of the boxed area in A-1. The black arrows indicate the proliferation of syncytiotrophoblast. (B) Histopathology showing p57kip2 immunohistochemistry in case No. 23. Expression of p57kip2 cannot be seen in the nuclei of cytotrophoblast cells and trophoblast stromal cells. (C) HE staining of a case of PHM (No. 18). C-1 shows a low magnification, and C-2 is a high magnification of the boxed area in C-1. Mild trophoblastic proliferation, stromal edema, and capillaries containing nucleated fetal red blood cells (yellow arrows) are observed. (D) Immunohistochemical examination of case No. 18 shows p57kip2 expression in the nuclei of cellular trophoblast cells (orange arrows) and villous interstitial cells (red arrow). HE: hematoxylin and eosin; CHM: complete hydatidiform mole; PHM: partial hydatidiform mole.

**Figure 2:**
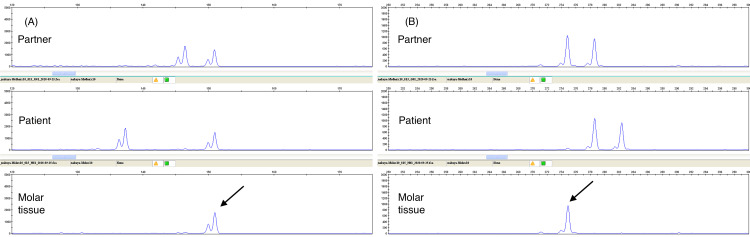
DNA genotyping analysis of CHM in case No. 23. (A) D8S1179. (B) D7S820. DNA genotyping analysis shows that all bands in the mole’s tissue were derived from the partner (black arrows). Genotyping data represent the partner, the patient, and the hydatidiform mole tissue. CHM: complete hydatidiform mole.

Figures 1A, 1B show the typical pathological findings of CHM in case No. 23. This case was judged pathologically as CHM, and genotyping showed homodisomy without maternal allele transmission. Figures 2A, 2B show the DNA genotyping results from case No. 23. Among the loci, D8S1179 and D7S820 are shown. The loci of the hydatidiform mole tissue were derived from the partner and not from the patient. These results indicated homodisomy, confirming the diagnosis of CHM.

**Figure 3:**
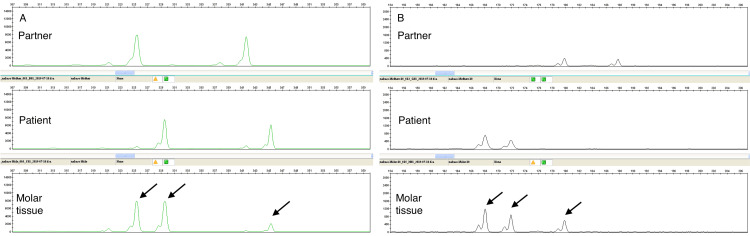
DNA genotyping analysis of PHM in case No. 18. (A) D2S1338. (B) vWS. Genotyping data represent the partner, the patient, and the hydatidiform mole tissue. At both loci, all villous tissues were of parental origin. The arrows pointing to the hydatidiform tissue indicate that it is triploid.

A case (No. 18) of typical PHM is shown in Figures 1C, 1D. This case was judged as PHM pathologically owing to the presence of p57kip2 expression, and genotyping results confirmed that the mole carried the maternal allele. An immunohistochemical examination showed p57kip2 expression in the nuclei of cellular trophoblast cells and villous interstitial cells. Figure 3 shows the DNA genotyping results from case No. 18. Among the loci, D2S1338 and vWS are shown. At the locus D2S1338, all villous tissues were of parental origin.

**Figure 4:**
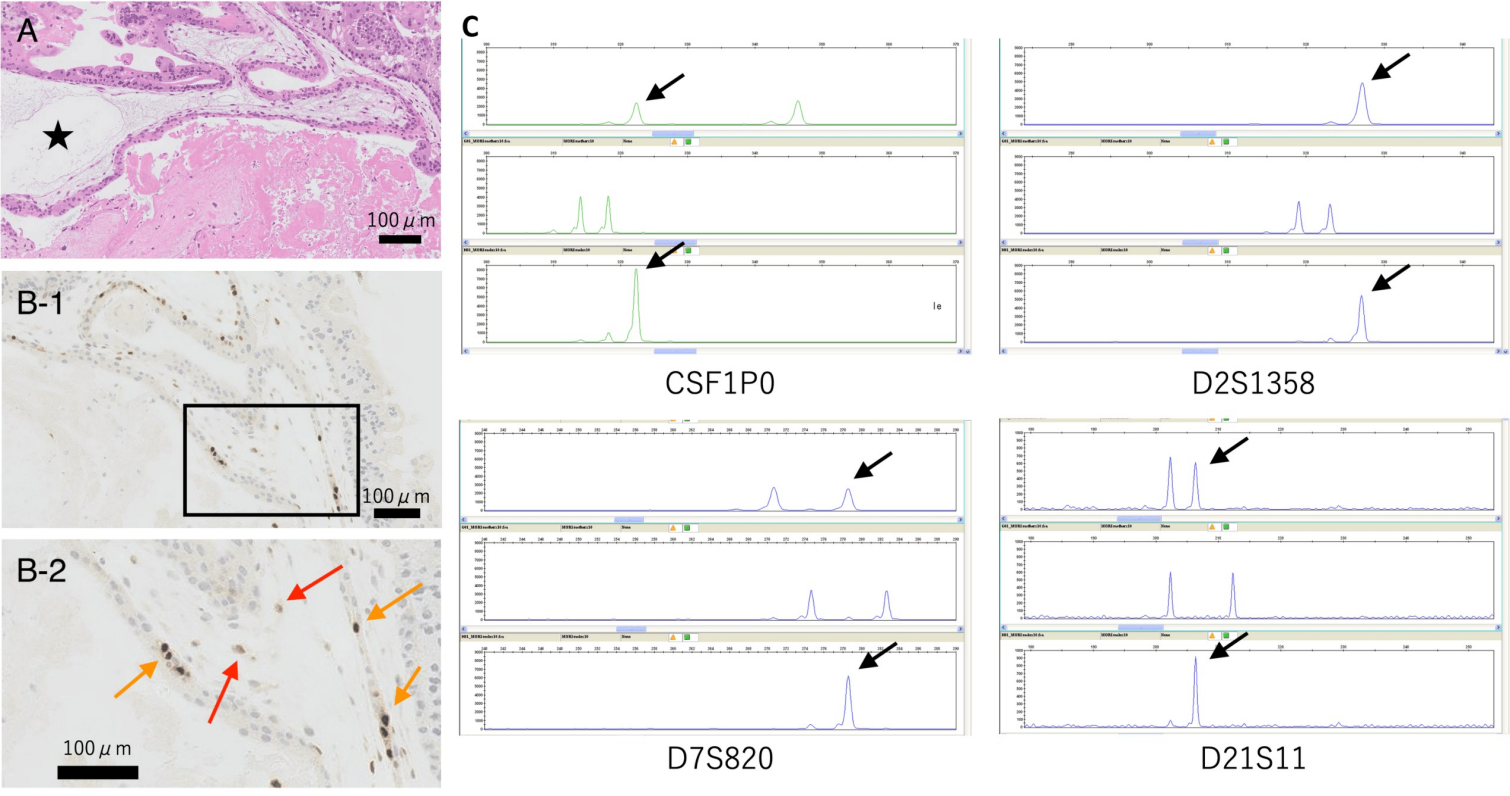
Discordant immunohistochemical and genotyping results of case No. 6. (A) HE staining shows edematous chorionic tissue (star). (B) An immunohistological examination. B-1 shows a low magnification, and B-2 is a high magnification of the boxed area in B-1. B-2 shows that some of the cytotrophoblast cells (orange arrows) and stromal cells (red arrows) are p57kip2-positive. (C) DNA genotyping analysis shows four DNA genotypes (CSF1P0, D2S1358, D7S820, and D21S11). The alleles of molar tissues indicated by black arrows were derived only from the partner, and the diagnosis of complete hydatidiform mole was made.

Figure 4 shows discordant immunohistochemical and genotyping results from case No. 6. A histological examination suggested that the mole was PHM owing to p57kip2 expression. However, a DNA genotyping analysis confirmed that the mole was CHM because the mother’s allele was not identified in the mole (Figure 4C). This patient was diagnosed as having PHM by the immunohistochemical examination, but as having CHM by the genotyping analysis.

Of the 19 cases of CHM diagnosed by DNA genotyping analysis in this study, blood samples were obtained from the partners of 16 patients (Table 1). Twelve (84.2%) cases showed homodisomy and three (15.8%) showed heterodisomy from paternal allele(s) in mole tissue. Of these 19 cases, only one case of GTD occurred in the paternal allele of a homodisomic mole. This case was classified as a clinical invasive mole because it scored 1 point using the International Federation of Gynecology and Obstetrics (FIGO) 2000 staging and risk factor scoring system for gestational trophoblastic neoplasia [17].

## Discussion

This study showed that a pathological immunological examination is not the ideal method of distinguishing CHM from PHM, when CHM is defined as occurring exclusively through paternal alleles. In Japan, there are only a limited number of facilities that use a DNA polymorphism analysis in conjunction with a pathological diagnosis for the differential diagnosis of hydatidiform mole. In this study, the positive predictive value and negative predictive value of the pathological diagnosis were 100% and 33.3%, respectively. These results suggest that p57kip2 immunohistochemistry is a useful tool, but not a perfect approach, and that adding DNA genotyping analysis of the chorionic tissue enables a more accurate diagnosis. These results are consistent with previous reports [4,11,12].

The collection of the proper part of hydatidiform mole tissue requires pathological experience and is essential for pathological/genetic examinations. According to previous studies, DNA genotyping analysis was not possible in 3.7% of cases because of difficulties in determining the genotype [18]. In this study, in some cases, tissues that were identified as hydatidiform moles and extracted were found to be decidua through genotyping. This finding indicates the importance of extracting DNA from the appropriate portion of the hydatidiform mole for a DNA polymorphism analysis and a pathological diagnosis.

We collected blood samples from the partners of the patients as much as possible to determine whether the CHM was due to homodisomy or heterodisomy. Recent reports have shown that the incidence of GTD is higher in heterodisomy than in homodisomy [14,15]. Kaneki et al. [19] performed DNA genotyping of villous tissues that were macroscopically diagnosed as hydatidiform mole, and analyzed 178 cases subsequently diagnosed as complete hydatidiform mole. They reported that among the 137 cases of homodisomy, 18 cases (13%) developed secondary complications, whereas among the 41 cases of heterodisomy, 10 cases (24%) developed such complications. These findings suggest that heterodisomy is associated with a higher risk of secondary complications, indicating that DNA genotyping, including that of the partner, provides valuable information for predicting clinical outcomes.

The limitations of this study include the small number of cases, but the fact that the cases were collected and analyzed at a single facility was an advantage in terms of standardizing the analysis method in this study.

## Conclusions

Distinguishing between CHM and PHM requires an accurate diagnosis because of the difference in the risk onset of GTD. Although there are technical limitations in the accuracy of a pathological immunological diagnosis and DNA genotyping, a more accurate diagnosis could be made by combining these two methods. On the other hand, regardless of the method used for differential diagnosis, appropriate sampling of the specimen is crucial and should be performed with greater care.

A diagnosis using DNA polymorphism analysis using various parts of the mole should be performed at more facilities. In addition, when CHM is diagnosed, there may be differences in the risk for developing GTD depending on a homo- or a heterodisomic hydatidiform mole. Therefore, follow-up studies of patients with homo- or heterodisomy should be carried out to clarify the differences in risk.

## Appendices

**Table 4:** Sizes and numbering for each DNA genotype.

	DNA genotype (sizes)														
No.	D8S1179	D21S11	D7S820	CSF1PO	D3S1358	TH01	D13S317	D16S539	D2S1338	vWS	TOPX	D18S51	Amelogenin	D5S818	FGA
1	131	198	263	317	113	169	220	268	310	165	229	238	108	144	227
2	133	201	265	319	125	172	221	271	312	168	231	279	112	145	228
3	135	202	267	321	126	173	224	272	314	172	233	283	160	148	229
4	137	205	269	322	129	176	225	273	316	176	234	287	-	149	231
5	139	206	271	323	133	177	227	275	317	180	238	290	-	153	241
6	141	209	273	325	134	180	229	276	318	184	241	291	-	157	244
7	142	210	275	326	138	181	231	278	320	185	242	294	-	161	245
8	143	211	276	327	-	184	232	279	321	188	243	295	-	165	-
9	146	212	277	329	-	187	234	280	322	-	246	297	-	141	-
10	147	214	278	330	-	-	236	282	324	-	247	298	-	-	-
11	150	215	279	331	-	-	237	283	325	-	251	300	-	-	-
12	151	217	282	333	-	-	238	284	326	-	273	302	-	-	-
13	152	227	284	334	-	-	242	285	328	-	-	304	-	-	-
14	155	-	285	335	-	-	243	287	329	-	-	306	-	-	-
15	156	-	286	337	-	-	245	288	330	-	-	307	-	-	-
16	159	-	-	339	-	-	247	291	332	-	-	308	-	-	-
17	-	-	-	-	-	-	249	168	333	-	-	310	-	-	-
18	-	-	-	-	-	-	217	-	334	-	-	311	-	-	-
19	-	-	-	-	-	-	-	-	336	-	-	315	-	-	-
20	-	-	-	-	-	-	-	-	338	-	-	316	-	-	-
21	-	-	-	-	-	-	-	-	341	-	-	324	-	-	-
22	-	-	-	-	-	-	-	-	342	-	-	328	-	-	-
23	-	-	-	-	-	-	-	-	345	-	-	320	-	-	-
24	-	-	-	-	-	-	-	-	346	-	-	-	-	-	-
25	-	-	-	-	-	-	-	-	348	-	-	-	-	-	-
26	-	-	-	-	-	-	-	-	349	-	-	-	-	-	-
27	-	-	-	-	-	-	-	-	353	-	-	-	-	-	-

**Table 5:** Data from all cases. The numbers in the cells for each DNA genotype correspond to the numbers in Table 4. For size-specific numbering, please refer to Table 4. CHM: complete hydatidiform mole, PHM: partial hydatidiform mole, hCG: human chorionic gonadotropin.

Case	Age (years)	Serum hCG levels (mIU/mL)	p57kip2	Pathological diagnosis	DNA genotyping	Sample	Locus																Homodisomy or heterodisomy
							D8S1179	D21S11	D7S820	CSF1PO	D3S1358	TH01	D13S317	D16S539	D2S1338	D19S433	vWS	TOPX	D18S51	Amelogenin	D5S818	FGA	
1	27	71608	Positive	PHM	PHM	Partner	1, 10	-	1, 11	5, 8	-	7	-	1, 12	12, 22	-	5, 6	6	3, 20	1, 2	4, 6	-	-
						Patient	5, 10	-	7, 11	5, 11	-	5, 7	-	1	1, 3	-	1, 4	1, 6	11, 13	1	4, 7	-	-
						Molar tissue	1, 5, 10	-	1, 11	5, 11	-	5, 7	-	1	3, 22	-	1, 6	1, 6	3, 13	1, 2	4	-	-
2	32	190388	Negative	CHM	CHM	Partner	3, 15	-	7, 11	8	-	7	-	9, 12	9, 18	-	3, 5	1, 6	6, 16	1, 2	-	-	Homodisomy
						Patient	8,10	-	5, 11	2, 5	-	7	-	9	20, 24	-	3, 4	1	4, 8	1	-	-	
						Molar tissue	15	-	11	8	-	7	-	9	18	-	5	6	6	1	-	-	
3	26	145313	Positive	PHM	Decidua	Partner	1, 3	-	-	2, 5	-	7, 8	-	1	9, 20	4	1	8, 11	-	1, 2	-	-	-
						Patient	3, 10	-	-	2	-	3	-	1, 15	6, 20	1, 3	1, 3	6, 13	-	1	-	-	-
						Molar tissue	3, 10	-	-	2	-	3	-	1, 15	6, 20	1, 3	1, 3	6, 13	-	1	-	-	-
4	29	40084	Negative	CHM	Decidua	Partner	1, 3	3, 8	3, 5	2, 11	-	3, 7	-	1, 12	9, 20	-	1, 3	1, 5	4, 8	1, 2	4, 6	-	-
						Patient	3, 13	8, 12	7, 11	8	-	5, 7	-	1, 3	9	-	5	1, 6	2, 13	1	2, 4	-	-
						Molar tissue	3, 13	8, 12	7, 11	8	-	5, 7	-	1, 3	9	-	5	1, 6	2, 13	1	2, 4	-	-
5	36	72367	Negative	CHM	CHM	Partner	1, 13	-	-	2	-	1, 7	-	3, 6	9, 24	-	1, 5	1, 6	6, 13	1, 2	-	-	Homodisomy
						Patient	5	-	-	2, 8	-	3, 5	-	6, 9	3, 9	-	1, 5	3, 5	13	1	-	-	
						Molar tissue	13	-	-	2	-	1	-	6	9	-	5	6	6	1	-	-	
6	50	146505	Positive	PHM	CHM	Partner	5, 8	3, 5	5, 11	8	2, 5	7	1, 18	1	9, 24	-	3, 4	1	4, 8	1, 2	5	-	Homodisomy
						Patient	8, 12	3, 9	7, 12	2, 5	2, 4	1, 7	6, 18	3, 17	3, 6	-	4	3, 6	8, 18	1	5, 9	-	
						Molar tissue	8	5	11	8	2	7	18	1	9	-	4	1	8	1	5	-	
7	30	141148	Positive	PHM	Decidua	Partner	3, 13	5	7	5	3	7	8, 18	1, 12	3	-	4, 5	3, 6	11	2, 3	4, 6	-	-
						Patient	10, 13	5	7	5, 8	2, 3	7	6, 8	3, 9	20, 24	-	4, 5	1, 6	6, 11	1	4, 9	-	-
						Molar tissue	10, 13	5	7	5, 8	2, 3	7	6, 8	3, 9	20, 24	-	4, 5	1, 6	6, 11	1	4, 9	-	-
8	34	23072	Negative	CHM	CHM	Partner	8, 13	5, 10	7, 11	5	2, 5	1, 7	4, 18	1, 9	9, 20	-	4, 8	1, 3	6, 23	1, 2	5	-	Heterodisomy
						Patient	3, 8	3, 5	7, 11	5, 7	2	1, 3	8	1, 3	9, 22	-	1	1	8, 11	1	5, 6	-	
						Molar tissue	3, 8	5, 10	7	5	5	7	4, 18	1, 9	9, 20	-	8	1, 3	23	1, 2	5	-	
9	44	340688	Negative	CHM	CHM	Partner	3, 10	3, 5	10	2, 8	-	1, 5	6, 8	1, 9	9, 22	-	3, 4	-	6, 18	1, 2	4, 6	3, 6	Heterodisomy
						Patient	1, 3	1, 9	7	5, 10	-	1, 3	5	3, 9	3, 9	-	1, 3	-	4, 8	1	2, 5	1, 4	
						Molar tissue	3	5	10	4, 8	-	5	6, 8	1, 9	9	-	3	-	18	1	4	3, 6	
10	40	153906	Negative	CHM	CHM	Partner	4, 14	7, 10	11	5	6	4, 8	2, 14	4, 9	22, 26	-	2, 6	4, 11	5, 21	1, 2	4, 7	2, 7	Homodisomy
						Patient	9, 16	4, 7	5, 11	8, 14	3, 6	2, 6	2	9, 12	12, 22	-	3, 5	4, 10	5, 10	1	5, 6	2, 5	
						Molar tissue	4	7	11	5	6	4	2	9	22	-	6	4, 11	21	1	7	2	
11	29	79360	Negative	CHM	CHM	Partner	4, 16	5, 7	11, 12	4, 8	1, 6	4, 8	2	8, 14	9, 15	-	5, 6	10	10, 12	1, 2	5, 7	-	Homodisomy
						Patient	9, 14	7	11, 12	11, 16	2, 4	2, 4	2, 12	3, 16	12, 20	-	4, 5	4, 10	12, 17	1	1, 6	-	
						Molar tissue	16	5	11	4	6	4	2	14	9	-	6	10	12	1	7	-	
12	35	451674	Negative	CHM	CHM	Patient	9, 12	3, 5	10	7	2, 6	8	4, 13	5, 8	5, 23	-	4	9	4	1	3, 7	-	-
						Molar tissue	6	7	7	7, 10	6	9	1	8	26	-	5	9	5	1	3	-	-
13	18	118811	Negative	CHM	CHM	Patient	2, 9	7, 10	3, 11	4, 7	-	4	1	8, 11	11, 14	-	2, 4	5, 10	7, 18	1	3, 6	-	-
						Molar tissue	2	5	3	10	-	8	4	6	14	-	2	10	7	1	3	-	-
14	28	180901	Negative	CHM	CHM	Partner	2, 9	4, 6	2, 9	6, 9	-	2, 8	15, 17	-	4, 10	-	2, 4	8, 10	10	1, 2	1, 5	-	Homodisomy
						Patient	2, 7	6, 11	9	9, 12	-	4, 6	7, 14	-	21	-	4, 6	2, 8	5	1	5, 6	-	
						Molar tissue	2	6	9	6	-	8	17	-	10	-	2	10	10	1	1	-	
15	25	302699	Positive	PHM	CHM	Partner	2, 16	6	6, 14	6, 9	-	8	-	7, 13	13, 16	-	5	2	5	1, 2	1, 7	-	Homodisomy
						Patient	2, 12	6	4, 6	6, 9	-	4, 8	-	7, 10	10, 19	-	2, 4	2	5, 7	1	5, 7	-	
						Molar tissue	16	6	6	6, 9	-	8	-	13	13	-	5	2	5	1	1, 7	-	
16	36	116319	Positive	PHM	CHM	Partner	2, 9	-	8, 15	4, 10	-	2, 8	-	2, 5	2, 11	-	2, 8	3, 9	12, 14	1, 2	-	-	Homodisomy
						Patient	2, 14	-	7, 12	7	-	4, 8	-	2, 11	17, 21	-	2, 5	3, 9	5, 12	1	-	-	
						Molar tissue	2	-	15	4	-	8	-	2	11	-	8	9	14	1	-	-	
17	33	83721	Negative	CHM	CHM	Partner	6, 14	8, 13	7, 11	-	4, 5	4, 6	-	2, 14	10	-	4, 8	8	10, 17	1, 2	1, 5	-	Homodisomy
						Patient	11, 14	5, 7	3, 11	-	4, 7	4, 6	-	5, 8	10, 25	-	6, 8	4	5, 19	1	5, 7	-	
						Molar tissue	14	-	11	-	4	4	-	2	10	-	8	8	10	1	5	-	
18	44	54369	Positive	PHM	PHM	Partner	9, 12	-	11, 12	4, 10	2, 4	2, 8	-	9	11, 22	-	5, 8	-	4, 7	1, 2	3, 5	-	-
						Patient	9, 12	-	7, 11	7, 13	2, 4	4, 8	-	3, 9	15, 23	-	2, 3	-	1	1	5, 7	-	-
						Molar tissue	9, 12	-	7, 11	7, 10	2, 4	4, 8	-	3, 9	11, 15, 23	-	2, 3 5	-	4, 22	1, 2	5	-	-
19	25	453838	Negative	CHM	CHM	Partner	7, 9	2, 7	3, 11	10, 13	-	6, 9	8, 11	-	4, 5	-	4, 5	-	12, 18	1, 2	3	-	Heterodisomy
						Patient	2, 12	5, 7	12	4	-	3, 11	21	-	2, 6	-	2, 6	-	7, 10	1	3, 5	-	
						Molar tissue	7	2, 7	3, 11	10, 13	-	9	8, 11	-	4	-	4	-	12, 18	1, 2	3	-	
20	29	49357	Negative	CHM	CHM	Partner	2, 10	-	5, 11	10	-	4, 8	4, 9	3, 5	21, 27	-	5, 6	10	6, 15	1, 2	5, 7	-	Homodisomy
						Patient	10	-	3, 5	7, 10	-	3, 4	2, 9	3, 5	5, 11	-	2, 6	4	7, 10	1	5, 6	-	
						Molar tissue	2	-	11	10	-	8	9	3	27	-	5	10	6	1	5	-	
21	53	299820	Positive	PHM	CHM	Patient	12, 15	-	3, 11	3, 9	-	2, 6	1, 6	8	11, 27	-	2	4, 9	6, 22	1	4, 6	-	-
						Molar tissue	15	-	11	3, 9	-	4	9	2	21	-	4	9	6	1	4	-	-
22	17	1047196	Negative	CHM	CHM	Patient	11, 14	-	13	3, 9	2, 4	4, 8	5, 10	7	1, 11	-	6	9	9	1	1, 3	-	-
						Molar tissue	11	-	10	9	4	4	8	7	7	-	6	12	9	1	6	-	-
23	33	75970	Negative	CHM	CHM	Partner	9, 12	5, 7	7, 11	1, 3	-	8	1, 6	2	8, 23	-	6	9	5	1, 2	1, 3	-	Homodisomy
						Patient	4, 12	8, 12	11, 12	7, 10	-	4, 8	3, 11	8, 10	8, 11	-	3, 4	7, 9	12, 14	1	3, 8	-	
						Molar tissue	12	5	7	1	-	8	6	2	8	-	6	9	5	1	1	-	
24	50	1128602	Negative	CHM	CHM	Partner	2, 11	5, 7	3, 11	9	-	8	8, 10	7, 13	7, 19	-	5, 6	9	-	1, 2	5, 8	-	Homodisomy
						Patient	4, 14	3, 7	7, 11	6, 15	-	8	8, 10	2, 7	16, 25	-	4	3, 9	-	1	3	-	
						Molar tissue	2	5	3	9	-	8	10	13	19	-	5	9	-	1	8	-	
